# A novel variant in *FN1* in a family with fibronectin glomerulopathy

**DOI:** 10.1038/s41439-019-0042-1

**Published:** 2019-02-27

**Authors:** Nabeel Aslam, Anshika Singh, Cherise Cortese, Douglas L. Riegert-Johnson

**Affiliations:** 1 Division of Nephrology and Hypertension, Mayo Clinic, Jacksonville, FL USA; 2 Department of Pathology, Mayo Clinic, Jacksonville, FL USA; 3 Divisions of Medical Genetics and Gastroenterology, Mayo Clinic, Jacksonville, FL USA

**Keywords:** Glomerular diseases, Genetic counselling

## Abstract

Glomerulopathy with fibronectin deposits (GFND) is a rare glomerular disorder. We report a 28-year-old male diagnosed with GFND by mass spectrometry on kidney biopsy tissue. Whole-exome sequencing (WES) identified that a previously undescribed *FN1* gene mutation (c.3051G > T, p.W1017C) was likely responsible for this patient’s fibronectin glomerulopathy. We discuss the implications of this novel variant of *FN1* and the importance of WES to identify a mutation in a gene of interest.

Glomerulopathy with fibronectin deposits (GFND) is a rare autosomal-dominant kidney disorder. Clinically, GFND is characterized by proteinuria, microscopic hematuria, hypertension, and progressive renal dysfunction. FN is a large dimeric glycoprotein that exists in a soluble (plasma) form and an insoluble (cellular) form. Excess deposition of the soluble (plasma) form is noted in GFND^1^. Thus far, two studies have shown an association between GFND and mutations in the FN 1 (*FN1*) gene in up to 50% of patients. This mutation is said to disrupt the FN–FN and FN–cell interaction by decreasing the affinity between the HEP II and Hep III domains in pFN, thereby reducing FN’s solubility and leading to deposition in the renal glomeruli^2,3^.

Histologically, GFND is characterized by a lobular glomerular architecture with mesangial expansion and obliteration of capillary loops due to the accumulation of an acellular material that is strongly periodic acid–Schiff positive. Immunofluorescence is typically negative, except for a few documented rare familial cases that were positive for immunoglobulin G (IgG) and C3^1^. The introduction of liquid chromatography–tandem mass spectrometry marked a significant advance in the diagnosis of GFND and other glomerular diseases. A definitive pathological diagnosis of GFND can be made using mass spectrometry through the identification of elevated FN expression in affected glomeruli^4^. Immunostaining using anti-FN antibodies is another established method to diagnose GFND^5^. We report a case of a 28-year-old male identified as having GFND with a novel variant of *FN1* using whole-exome sequencing (WES).

A 27-year-old male was referred to our institution for a second opinion for the management of glomerulonephritis. He was asymptomatic on presentation without any urinary complaints, leg swelling, or rash. His renal history goes back to the age of 17 years, when he was found to have microscopic hematuria and nephrotic-range proteinuria and underwent a renal biopsy. His renal biopsy findings were reported as “mesangial proliferative glomerulonephritis,” and no additional details were available. He had been treated with prednisone without any improvement in proteinuria, followed by mycophenolate mofetil and tacrolimus. There was a gradual decline in his glomerular filtration rate (GFR) while on immunosuppressant medicines, and he was referred to our center for further management. His family history was significant for his biological father, who received a diagnosis of “glomerulonephritis” at 23 years of age requiring kidney transplant at age 54 years. The patient was normotensive with normal physical exam findings except trace lower-extremity edema. On presentation, his serum creatinine concentration was 2.1 mg/dL, with an estimated GFR of 38 ml/min, serum potassium of 5.3 mmol/l, and serum uric acid of 11.3 mg/dl. Urine analysis showed 1+ protein and bland urinary sediment. The random urine protein-to-creatinine ratio was 2.6. Hepatitis serology and complement levels were normal.

A repeat renal biopsy showed a lobular architecture in the glomeruli as well as segmental sclerosis. The mesangium was increased, and capillary loop obliteration was present. Double contours were present on silver staining (Fig. 1a). Congo red staining was negative. Immunofluorescence showed 2+ granular mesangial staining of IgM, suggestive of Ig trapping. Electron microscopy showed two glomeruli, both with a normal basement membrane thickness. There were abundant, finely granular 6–10 nm fibrillary deposits in subendothelial and mesangial locations causing capillary loop obliteration (Fig. 1b). These fibrils were suggestive of FN, and the deposits were examined by liquid chromatography–tandem mass spectrometry, which confirmed the presence of FN (Fig. 1c, d). The patient’s diagnosis was classified as GFND, and immunosuppressive medicines were discontinued.

**Fig. 1:**
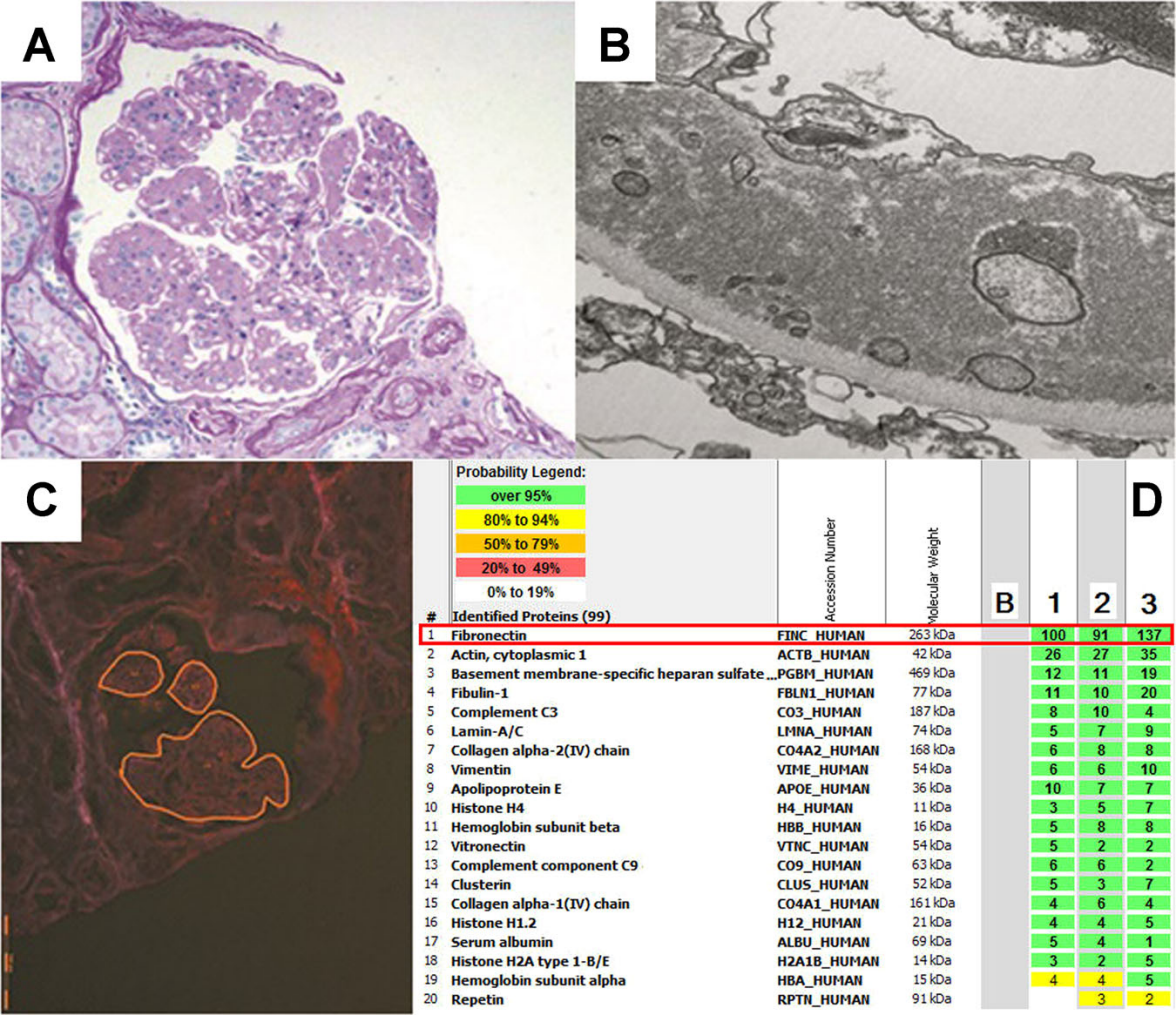
**a** Glomerulus with a lobular architecture and mesangial expansion with capillary loop obliteration (periodic acid–Schiff, ×20) **b** Electron microscopy of the glomerular basement membrane showing subendothelial finely granular fibrillary deposits (×23,000 direct magnifications). **c** Glomerulus microdissected for proteomic analysis with lobular architecture (Congo red, ×20). **d** List of proteins identified by proteomic analysis in three different glomeruli (1–3). Fibronectin is the most abundant protein in all three samples. “B” represents a blank (control) sample. The numbers indicate peptide spectra identified for each protein in each sample

WES was completed in our patient, his brother, and his father. Testing was conducted by Ambry Genetics in Aliso Viejo, CA. A variant in *FN1* (c.3051G>T, p.W1017C) was identified. Co-segregation analysis was completed showing that both our patient and his affected father harbor the mutation, while his unaffected brother does not.

GFND is characterized by massive glomerular deposits of FN, which causes disruption of the glomerular architecture and the filtration barrier, leading to glomerular proteinuria, reduction of the GFR and, eventually, end-stage renal disease (ESRD)^6^. Based on a clinical observation of its autosomal-dominant pattern of inheritance and age-related penetrance^7^, GFND has been thought to be a classical autosomal-dominant Mendelian disorder. Linkage to the *FN1* gene locus has been reported in several Italian and Japanese pedigrees. In 2008, Castelletti et al. sequenced *FN1* in 15 unrelated pedigrees and found 3 heterozygous missense mutations (W1925R, L1974R, and Y973C) that co-segregated with the disease in 6 pedigrees. The mutations affected two domains of FN: the Hep-II domain for W1925R and L1974R and the Hep-III domain for Y973C^2^. In a more recent large-scale analysis of 12 GFND families, 6 *FN1* mutations were detected, with 5 of them being novel (p.Pro969Leu, p.Pro1472del, p.Trp1925Cys, p.Lys1953_Ile1961del, and p.Leu1974Pro). p.Pro1472del was localized in the integrin-binding domain of FN, while the other mutations were in heparin-binding domains^3^. The *FN1* gene located at 2q34 encodes FN, which is a plasma protein that binds cell surfaces, collagen, heparin, DNA, actin, and fibrin. The mutation in the *FN1* gene has a deleterious effect on FN–cell interaction and FN fibrillogenesis^2^.

We performed WES with the hope of finding a causative mutation, whether in *FN1* or in another gene, for this family. In our proband, WES detected a heterozygous variant in *FN1* (c.3051G>T, p.W1017C). The results were submitted to Clinvar (NM_212482.2) by Ambry in 2013. Co-segregation analysis revealed that the affected father also has the heterozygous alteration, while the unaffected brother does not have the alteration (Fig. 2). This variant has not been reported to be a pathogenic or a benign variant in GFND. The W1017 amino acid position is highly conserved among vertebrate species. The probability of an alteration with potential deleterious effects is higher for evolutionarily conserved positions^8^. The p.W1017C alteration is located in exon 20 of the HEP-III heparin-binding domain of the *FN1* gene. The tryptophan at codon 1017 is replaced by cysteine, which is an amino acid with highly dissimilar properties. PolyPhen-2 deemed the mutation to be probably damaging, with a score of 1, and SIFT predicted it to be deleterious, with a score of 0.05. Considering all of the above evidence, this *FN1* mutation (W1017C) was judged to be pathogenic.

**Fig. 2:**
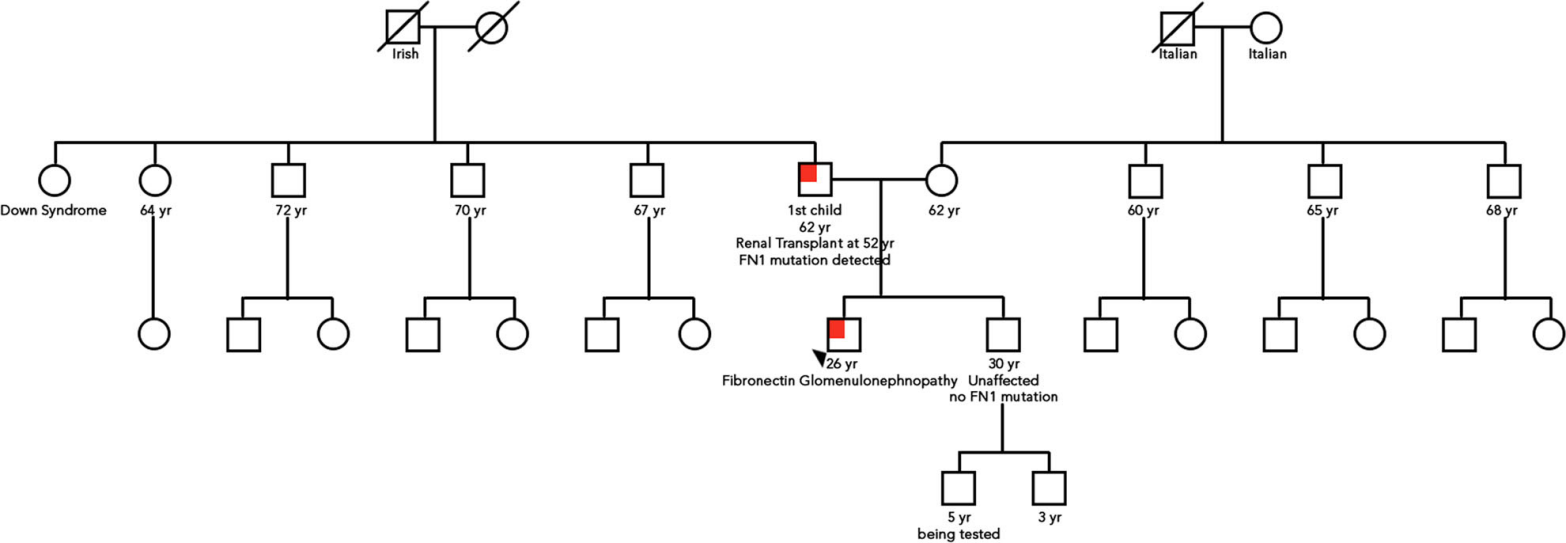
Family pedigree: the proband is indicated with an arrowhead

GFND appears to be genetically heterogeneous, with many families not having an *FN1* mutation. Identification of the *FN1* mutation using WES allows our patient the option of conceiving a child without the familial *FN1* mutation via preimplantation genetic testing (PGT). With this newly documented *FN1* mutation, PGD will give our patient and his wife the ability to prevent future generations from developing GFND. After the results were reported, the couple requested referral to a clinic with expertise in PGT.

While there is currently no effective treatment for GFND, management is aimed at preserving renal function and reducing proteinuria by optimizing blood pressure control, use of renin–angiotensin–aldosterone system blockers, and avoidance of potentially nephrotoxic agents. Since it is not an immune disease, immunosuppressive therapy is not warranted^6^. The options of dialysis and renal transplant are considered in patients who develop ESRD^9^. However, GFND can recur in the transplanted kidney^10^.

In summary, *FN1* (p. W1017C) is a novel pathogenic variant and was responsible for this patient’s GFND, and the exact mechanism of *FN1* mutation causing GFND merits further investigation.

## Data Availability

The relevant data from this Data Report are hosted at the Human Genome Variation Database at 10.6084/m9.figshare.hgv.2537.
